# The early diagnosis of Alzheimer's disease: Blood‐based panel biomarker discovery by proteomics and metabolomics

**DOI:** 10.1111/cns.70060

**Published:** 2024-11-21

**Authors:** Yun Dong, Xun Song, Xiao Wang, Shaoxiang Wang, Zhendan He

**Affiliations:** ^1^ College of Pharmacy Shenzhen Technology University Shenzhen China; ^2^ Department of Pharmacy Shenzhen People's Hospital (The Second Clinical Medical College, The First Affiliated Hospital, Jinan University, Southern University of Science and Technology) Shenzhen China; ^3^ School of Pharmaceutical Sciences, Health Science Center Shenzhen University Shenzhen China

**Keywords:** Alzheimer's disease, biomarkers, metabolomics, plasma, proteomics, serum

## Abstract

Diagnosis and prediction of Alzheimer's disease (AD) are increasingly pressing in the early stage of the disease because the biomarker‐targeted therapies may be most effective. Diagnosis of AD largely depends on the clinical symptoms of AD. Currently, cerebrospinal fluid biomarkers and neuroimaging techniques are considered for clinical detection and diagnosis. However, these clinical diagnosis results could provide indications of the middle and/or late stages of AD rather than the early stage, and another limitation is the complexity attached to limited access, cost, and perceived invasiveness. Therefore, the prediction of AD still poses immense challenges, and the development of novel biomarkers is needed for early diagnosis and urgent intervention before the onset of obvious phenotypes of AD. Blood‐based biomarkers may enable earlier diagnose and aid detection and prognosis for AD because various substances in the blood are vulnerable to AD pathophysiology. The application of a systematic biological paradigm based on high‐throughput techniques has demonstrated accurate alterations of molecular levels during AD onset processes, such as protein levels and metabolite levels, which may facilitate the identification of AD at an early stage. Notably, proteomics and metabolomics have been used to identify candidate biomarkers in blood for AD diagnosis. This review summarizes data on potential blood‐based biomarkers identified by proteomics and metabolomics that are closest to clinical implementation and discusses the current challenges and the future work of blood‐based candidates to achieve the aim of early screening for AD. We also provide an overview of early diagnosis, drug target discovery and even promising therapeutic approaches for AD.

## Graphical abstract

Because of CSF biomarkers identified by neuroimaging techniques in the middle or late stages of AD, blood is considered as the novel biomarker resource for the early diagnosis of AD. The accurate alternations in blood could predict AD. This paper discusses potential blood‐based biomarkers identified by proteomics and metabolomics and future research direction for AD prediction, and provide an overview of early diagnosis and even promising therapeutic approaches for AD.

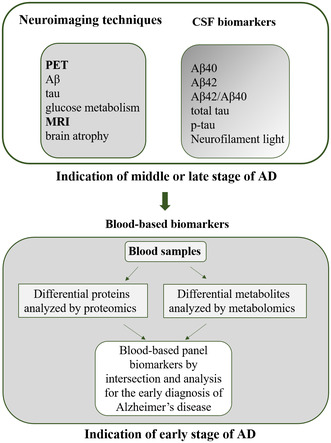

## 1 INTRODUCTION

Alzheimer's disease (AD), which is the prevalent cause of dementia accompanied by progressive memory impairments and irreversible cognitive dysfunctions, affects 34 million aging people, and this population is projected to triple by 2050.^1^ The pathophysiological characteristics of AD typically include a loss of neurons, amyloid‐beta (Aβ) aggregation, hyperphosphorylated tau‐induced neurofibrillary tangles, and a decrease in acetylcholine in clinical trials. AD progress includes a continuum of preclinical, prodromal, and dementia stages. A therapy for AD is defined as an intervention in the clinical progression of AD by interfering with the underlying pathophysiological mechanisms.

A diagnosis of AD is mostly based on the results of neuropsychological assessments and neuroimaging.^2^ A definitive diagnosis of AD requires postmortem evaluation of brain tissue or biomarkers in cerebrospinal fluid (CSF); however, neuroimaging techniques accompanied by several relatively clinical criteria can identify AD in living patients.^3^, ^4^ For instance, mesial temporal lobe atrophy has been identified by magnetic resonance imaging (MRI) in clinical trials. Several CSF biomarkers have been used for clinical diagnosis of AD, such as decreased levels of Aβ1‐42 identified on a positron emission tomography (PET) scan, increased levels of total tau on MRI, high levels of tau phosphorylated at threonine 181 (p‐tau181) on MRI.^5^, ^6^, ^7^ However, these diagnostic approaches have a marked shortcoming in that a diagnosis can only be made after the onset of obvious symptoms based on neuropsychological assessment.^2^, ^8^ Another limitation is the complexity attached to a limited access and the perceived invasiveness, which restricts the use of these biomarkers to specialized centers.^9^, ^10^ Therefore, AD prediction among individuals still poses an outstanding challenge. This context energizes the field to develop novel biomarkers to identify individuals at the earliest preclinical stages of AD and thereby facilitate early intervention and delay or even prevent the onset of obvious phenotypes. Blood is more accessible from the population than tissue or CSF, and its relative inexpensiveness and less‐invasiveness and substitutability facilitate the sampling of large cohorts. It is also more suitable for repeated sample collection for longitudinal assessment. It is important to note that various substances in the blood that may catalog the entire molecular information are vulnerable to the pathophysiological mechanism of AD.^11^, ^12^, ^13^, ^14^ Plasma proteins could be used to study organ aging to predict diseases, such as AD.^15^ Accordingly, blood is increasingly recognized as a promising target for the discovery of biomarkers to predict AD.

Based on advances in high‐throughput techniques, a systematic biological paradigm that includes various omics and bioinformatics has been globally applied for the etiological and pathological explorations of complex diseases such as diabetes and cancers with accurate and rigorous computational modeling.^16^, ^17^, ^18^ Because the application of omics, including proteomics and metabolomics, based on high‐throughput platforms can show accurate alterations in molecular levels such as protein levels and metabolite levels, it has been also increasingly used to identify biomarkers for prediction of cognitive performance, conversion from mild cognitive impairment (MCI) to AD dementia or for differential diagnosis. For instance, Zetterberg^19^ demonstrated a large volume of data from AD patients and healthy populations of the same age using high‐throughput technology platforms. Moreover, Kim et al.,^20^ found that specific phospholipase c gamma‐1 single‐nucleotide variants (SNV) could predict AD by high‐throughput screening. Researchers have adopted high‐resolution mass spectrometry,^21^ highly sensitive multiplexed immunoassay platforms,^22^, ^23^ and aptamer‐based assay platforms^24^, ^25^ for proteomic profiling of blood and discovery of AD protein signatures.

**FIGURE 1:**
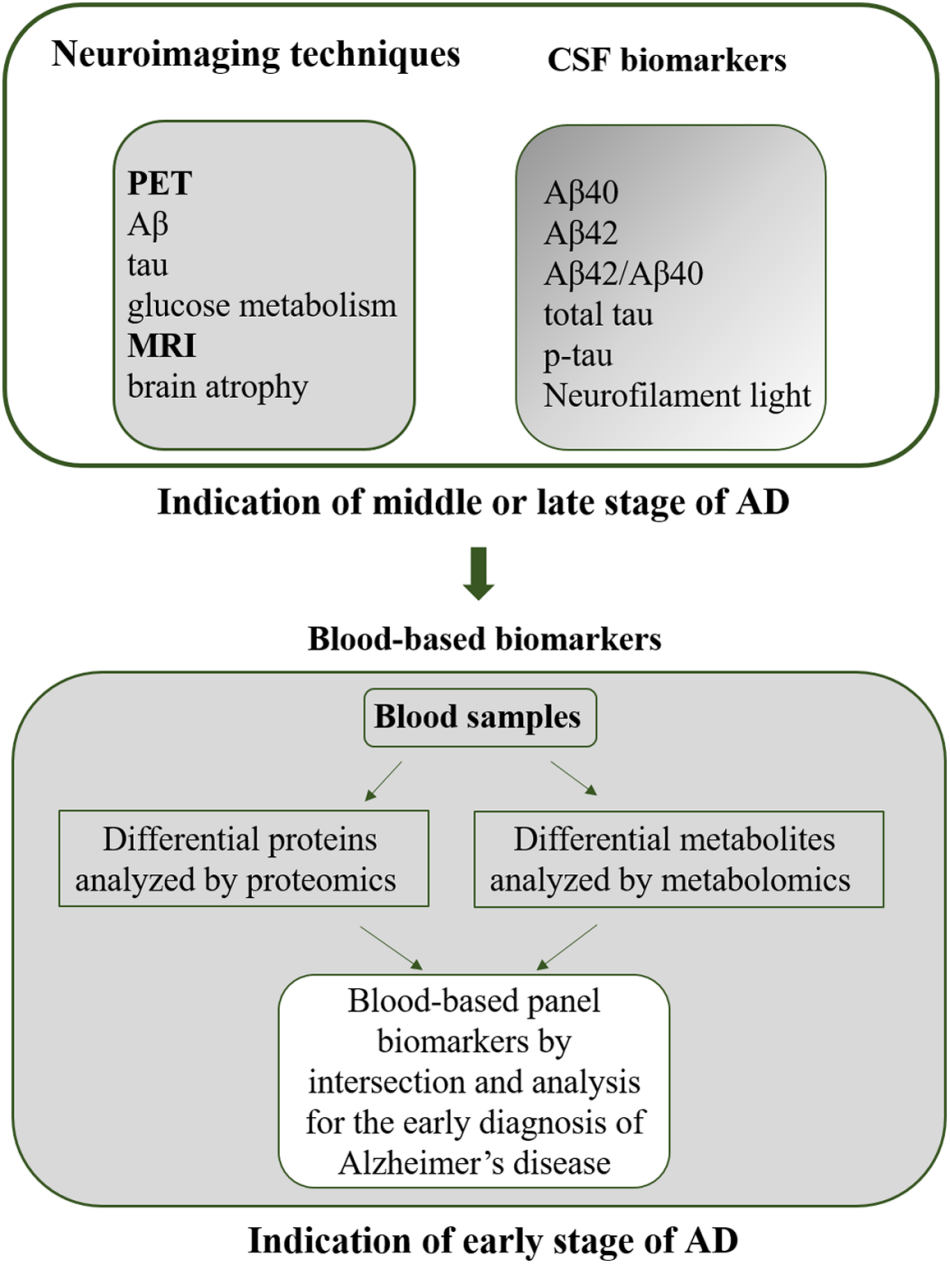
A schematic overview of established and candidate blood‐based panel biomarker discovery for Alzheimer's disease prediction by proteomics and metabolomics.

Proteomics and metabolomics have been widely used to identify candidate biomarkers in brain tissues and biofluids for diagnosis of AD during AD progression.^22^, ^23^, ^26^, ^27^, ^28^ Because blood likely contains an entire proteome and metabolites are a reliable target source, an accumulating body of studies has recently explored the potential of blood‐based biomarkers to provide information on pathological processes of AD and identified blood‐based candidate biomarkers that may predict AD. This review includes an overview of potential blood‐based biomarker candidates that have been analyzed by proteomics and metabolomics (Figure 1), which are moving the field toward prognosis, early diagnosis, and intervention, and even toward promising therapeutic approaches for AD.

## 2 CLASSIC BLOOD‐BASED BIOMARKERS ANALYZED BY PROTEOMICS FOR AD PREDICTION

The CSF biomarkers, including Aβ1‐42, total tau (t‐tau), and phosphorylated tau (p‐tau), have been shown good sensitivity and specificity for differentiating AD patients from healthy controls^29^, ^30^ and succeed as a diagnosis for AD. Because blood‐based tests are widely available, minimally invasive, and relatively inexpensive, they can serve as efficient prescreening tools in a diagnostic process of AD.^31^ Previous studies have demonstrated alternations of proteomic profiles in blood samples of AD subjects,^32^, ^33^ implying the feasibility of blood‐based biomarkers. The development of blood‐based biomarkers is facilitated by advances in not only targeted approaches but also omics technologies.^34^, ^35^

### 2.1 Aβ levels

Aβ levels could be an important target candidate for the prediction of AD. Aβ levels in blood were significantly related to AD genetic variants.^36^ The early results described by Olsson et al.^37^ revealed that neither Aβ1‐42 nor Aβ1‐40 in plasma discriminated AD subjects from control subjects, as rated by random‐effects meta‐analysis. Olsson and colleagues found that the levels of plasma Aβ1‐42 did not differ significantly between patients with AD from control subjects in 22 AD (*n* = 1872) and 20 control cohorts (*n* = 3855) (average ratio 1.04, 95% CI 0.96–1.12, *p* = 0.32). Plasma or serum concentration of Aβ1‐40 was not significantly different in 21 AD and 19 control cohorts comprised of patients with AD (*n* = 1661) and control subjects (*n* = 3668) (average ratio 1.04, 0.98–1.11, *p* = 0.17). Moreover, Pannee and colleagues also investigated blood‐based Aβ1‐38, Aβ1‐40, and Aβ1‐42 with an antibody‐free CSF reference multiple/selected reaction monitoring (MRM) in a small cohort of nine patients with AD and 10 control subjects and observed no significant differences between the two groups (*p* = 0.13, 0.49, and 0.13, respectively).^38^ Furthermore, the group detected Aβ variants (Aβ1‐15, Aβ1‐17, Aβ1‐19, Aβ1‐20, Aβ1‐33, Aβ1‐34, Aβ5‐40, Aβ1‐37, Aβ3‐40, and Aβ1‐39) using immunoprecipitation and matrix‐assisted laser desorption/ionization mass spectrometry MS and did not find differences between samples from patients with AD and control subjects. These findings indicate that blood‐based Aβ peptides may not be candidate biomarkers for AD diagnosis.

Kim and colleagues, however, provided an accurate quantitation of plasma Aβ levels with an improved antibody‐free method, selected reaction monitoring MS, which could differentiate patients with AD from control subjects.^39^ Recent findings regarding the relationship between plasma Aβ levels and AD pathogenesis are contradictory. One study showed that of the 392 patients with dementia 289 were diagnosed with AD (the ratio of Aβ1‐42/Aβ1‐40: 0.55) and 54 with vascular dementia (the ratio of Aβ1‐42/Aβ1‐40: 0.49), suggesting that an increased ratio of Aβ1‐42/Aβ1‐40 in the plasma was related to an increased risk of developing AD.^40^ On the contrary, several studies reported that a reduction in the plasma Aβ1‐42/Aβ1‐40 ratio increased the risk.^12^, ^41^, ^42^, ^43^ Notably, PET measures of brain amyloid burden do show an association between a reduced plasma Aβ1‐42/Aβ1‐40 ratio and an increased brain amyloid load,^43^ which indicates that a decreased plasma Aβ1‐42/Aβ1‐40 ratio could increase the risk of AD. Kaneko et al.^44^ further measured the plasma Aβ levels in 22 patients with AD and 40 control subjects (those with MCI and healthy subjects) and verified a significant reduction in the Aβ1‐42 and Aβ1‐42/Aβ1‐40 ratios in individuals with AD compared with the control subjects, and the reduced Aβ1‐42/Aβ1‐40 ratio could differ patients with AD from control subjects with a high 92.5% sensitivity and 95.5% specificity. Nakamura and colleagues demonstrated that the reduced ratio of Aβ1‐42/Aβ1‐40 could differ patients with AD from controls under the receiver operating characteristic curves (area under the curve: AUC) in both data sets (discovery, 96.7%, *n* = 121 and validation, 94.1%, *n* = 111) with a high accuracy approximately equal to 90%.^12^ In addition, a preclinical study by Chouraki et al.^41^ revealed that a low plasma Aβ1‐42/Aβ1‐40 ratio was associated with an increased risk of developing dementia or incident AD. Likewise, measurement by immunoprecipitation coupled with MS‐based PCA assays showed a reduction in the plasma Aβ1‐42/Aβ1‐40 ratio in patients with AD with approximated 90% accuracy.^12^, ^45^ Startin et al.^46^ also demonstrated that the plasma Aβ1‐42/Aβ1‐40 ratio is higher in control individuals than in patients with AD. Chatterjee et al. showed that the plasma Aβ1‐42/Aβ1‐40 ratio was significantly lower in individuals with preclinical AD (cognitively unimpaired Aβ+), prodromal AD (MCI Aβ+), or AD.^47^

These studies suggested that a reduction in the Aβ1‐42/Aβ1‐40 ratios in plasma could be used for the diagnosis of AD. However, the diagnostic value of plasma Aβ1‐42/Aβ1‐40 ratios does not surpass that of CSF Aβ1‐42 used alone. Therefore, additional investigations are needed to show whether plasma Aβ1‐42/Aβ1‐40 ratios may be reflective of AD processes.

### 2.2 Tau levels

Evidence showed that plasma p‐tau biomarkers were strongly associated with autopsy‐confirmed AD on PET.^48^ Increasing reports have demonstrated that plasma p‐tau levels could differentiate cases with AD pathology from Aβ‐negative controls.^34^, ^49^, ^50^, ^51^, ^52^

Plasma p‐tau181 could be a reliable biomarker for the prediction of AD. The level of p‐tau181 has been found to increase in plasma in preclinical AD.^53^, ^54^, ^55^ A literature analyzed by Thijssen and colleagues^56^ showed that plasma p‐tau181 concentrations were increased by 3.5 folds in patients with AD compared to control cohorts. Importantly, the increased levels of p‐tau181 in plasma could differ patients with AD from non‐AD cohorts with a high accuracy (AUC = 0.97, 95% CI = 94.1–100%). Rodriguez and colleagues also showed that the significant increases of plasma p‐tau181 occurred approximate 8 years prior to death in patients with AD and till later plateauing.^54^ In contrast, non‐AD control cohorts up until death exhibited non‐significant increases of p‐tau181 in plasma. These studies suggest that plasma p‐tau181 could be an available diagnostic indicator for cohorts in the early stage of AD. Karikari and colleagues have demonstrated that plasma p‐tau181 gradually increased along the AD processes and also elaborated that plasma p‐tau181 could discriminate patients with AD dementia with high accuracy from Aβ‐negative young adults (AUC = 0.99) and cognitively unimpaired older adults (AUC = 0.90–0.98 across cohorts).^53^ Notably, the levels of plasma p‐tau181 not only highly predict tau pathologies but also specifically reflect amyloid plaque pathology in the discovery cohorts (*n* = 36, AUC = 0.98), identifying Alzheimer's disease from other neurodegenerative disorders.^57^ A recent study also reported that plasma p‐tau181 was specifically associated with Aβ‐positive neurodegeneration among participants with AD dementia.^58^ Given that, these findings suggest that plasma p‐tau181 could be used as a scalable and accessible tool for screening and diagnosis of AD.

Plasma p‐tau217 is thought to be an independent candidate biomarker for AD prediction. Recently, increasing studies showed that the increased levels of p‐tau217 in plasma were highly correlated with amyloid plaque pathology in the cohorts with AD patients.^48^, ^55^, ^57^ For instance, Barthélemy and colleagues found that plasma p‐tau217 was a highly accurate and specific predictor for AD pathology in the discovery cohort (*n* = 36, AUC = 0.99) and in the validation cohort (*n* = 92, AUC = 0.92).^57^ Palmqvist and colleagues have also reported that plasma p‐tau217 differentiated preclinical AD patients from Aβ‐negative controls with a high accuracy (*n* = 301, AUC = 0.90) and identified prodromal AD patients (AD with MCI) from Aβ‐negative MCI cohorts (*n* = 178, AUC = 0.96).^52^ Moreover, plasma p‐tau217 accurately distinguished Aβ‐PET+/tau‐PET– cognitively unimpaired participants from Aβ‐PET−/tau‐PET– cognitively unimpaired participants with high accuracy (AUC = 0.83).^59^ Longitudinal measures showed significantly accelerated levels of plasma p‐tau217 in preclinical and prodromal AD patients compared to Aβ‐negative unimpaired adults (*p* < 0.001) and Aβ‐negative MCI patients (*p* < 0.001).^60^ Also, the authors found that plasma p‐tau217 was not only highly correlated with CSF p‐tau217 and Aβ burden for all cohorts but also precisely differed AD dementia from non‐AD neurodegenerative disorders. These findings suggest that plasma p‐tau217 could be used to predict early AD. Interestingly, plasma p‐tau217 could identify Alzheimer's co‐pathology in patients with Lewy body disease.^61^ The associations of longitudinal plasma p‐tau217 with progressive neurodegeneration and cognitive decline were largely congruent with p‐tau181, and plasma p‐tau217 concentration was increased by both Aβ plaques and tau tangles.^60^ Totally, these results suggest that plasma p‐tau217 could be another more accurate preclinical biomarker that differentiates patients with AD from all control cohorts and could be developed an early diagnosis of AD. Plasma p‐tau217 as a potential biomarker for AD prediction has been validated in research setting. Next, the clinical results of plasma p‐tau217 would be collected and must be consistent with research results at the level of individual patients. Furthermore, clinical evaluation of plasma p‐tau217 for AD prediction should focus on establishing large databases.

An ultrasensitive single molecule array showed that plasma p‐tau231could be as a novel biomarker for incipient AD pathology.^34^ Ashton and colleagues found that plasma p‐tau231 was able to identify patients with AD from Aβ‐negative older adults (AUC = 0.92–0.94) and non‐AD neurodegenerative disorders (AUC = 0.93), as well as Aβ‐negative MCI patients (AUC = 0.89) in different independent cohorts (*n* = 588). Also, plasma p‐tau231 was highly correlated with CSF p‐tau231 and tau pathology. Additionally, plasma p‐tau231 accurately differed in AD neuropathology (AUC = 0.99) in a neuropathology cohort. Hence, if enough investigations confirm plasma p‐tau231 to identify patients with AD in both research and clinical settings, plasma p‐tau231 can be as a biomarker for AD prediction.

Plasma t‐tau is also thought to be a result of neuronal damage that causes tau release from the brain, and plasma t‐tau levels are associated with the severity of neurodegeneration.^62^ Meta‐analysis has shown levels of plasma t‐tau to be increased in patients with AD compared with control cohorts (*p* = 0.02).^37^ Two independent studies have also demonstrated that elevated plasma t‐tau could reflect a brain disorder in patients with AD dementia.^41^, ^63^ Fossati and colleagues^41^ corroborated that plasma t‐tau was an independent sign, as plasma t‐tau is correlated with neither CSF total tau nor CSF phosphorylated tau, which was supported by previous studies.^64^ However, it is interesting to note that the addition of plasma t‐tau to the CSF total tau or CSF phosphorylated tau leads to a significant improvement in diagnostic accuracy.^41^ Also, patients with AD showed a substantial overlap in levels of plasma t‐tau with cognitively unimpaired elderly subjects based on the large Alzheimer's Disease Neuroimaging Initiative (ADNI) and Biomarkers For Identifying Neurodegenerative Disorders Early and Reliably cohorts.^63^ These findings suggest that plasma t‐tau partly reflects AD and could not be an appropriate biomarker for AD prediction. In addition, a novel blood‐based biomarker, brain‐derived tau level in serum was significantly increased in AD participants and could separate AD cohorts from controls using an immunoassay.^65^

Given that, although plasma p‐tau181, p‐tau217, and p‐tau231 could be an independent biomarker for predicting AD, more research validation and clinical validation must be completed. In addition, the clinical evaluation should focus on establishing large databases. For instance, the concentrations of plasma p‐tau181, p‐tau217, or p‐tau231 of individual patients with AD alongside their clinical characteristics should be included.

### 2.3 Apolipoprotein E levels

Apolipoprotein E (ApoE) has three major isoforms, ApoE2, ApoE3, and ApoE4, and plays a prevalent role in the brain. Among these three isoforms, ApoE4 is the most important genetic risk factor for AD^66^ and could be an interesting blood‐based biomarker. Han et al.^67^ measured total ApoE in serum using several orthogonal methods coupled with MRM and observed lower serum ApoE levels in individuals with AD than in control subjects. Two other studies found no differences in plasma ApoE in patients with AD and control subjects (^68^;). Inconsistent results of ApoE levels in blood samples from patients with AD were also reported in several immunoassay‐based studies.^69^ Evidence has shown that total ApoE levels in blood differ among carriers of different ApoE genotypes.^68^, ^70^ These results render ApoE quite unsuitable as a candidate biomarker for the detection of AD. Although several groups have reported successful identification of ApoE isoforms in blood by quantifying allele‐specific peptides with 100% concordance to classical genotyping,^70^ the development of blood‐based ApoE as a candidate biomarker for AD prediction remains difficult.

## 3 BLOOD‐BASED BIOMARKER PANELS IDENTIFIED BY PROTEOMICS FOR AD PREDICTION

Blood‐based biomarker panels with acceptable clinical performance are selected as the optimal approach for AD prediction.^71^ A substantial number of proteins related to the prognosis or diagnosis of AD have been identified from blood with the use of proteomic approaches. Several studies have shown the efficacy of multivariate panels of proteins to differentiate cases from control cohorts with great accuracy and specificity (Table 1).

**TABLE 1:** Discovery of based‐blood protein panels for diagnosis of Alzheimer‘s disease.

Protein panels			Reference
Plasma‐based proteins		ANG‐2, CCL5, CCL7, CCL18, CXCL8, EGF, G‐CSF, GDNF, ICAM‐1, IGFBP‐6, IL‐1α, IL‐3, IL‐11, M‐CSF, PDGF‐BB, TNF‐α, TRAIL‐R4	^33^
		IL‐1α, IL‐3, EGF, TNF‐α, G‐CSF	^72^
		AAT, A2Macro, ApoE, complement C3	^24^
		A2Macro, AAT, complement C3, ApoE, A1Macro, PPP	^76^
		CFH, α‐2 M	^32^
		Clusterin, TTR, cystatin C, A1AcidG, ICAM1, CC4, PEDF, A1AT, RANTES, ApoC3	^79^
		Clusterin	^24^
		Apolipoprotein A‐1, α‐2‐HS‐glycoprotein, apolipoprotein A‐4, fibrinogen gamma chain	^75^
		OSM, MMP‐9, HAGH, CD200, AXIN1, uPA	^23^
		APP, NfL, APBB3, GPSM2, REST, DNAH10, FHAD1, NGN2.	^82^
		A2Macro, ERAP2, CDH5, ACE, LGALS3BP	^78^
		Aβ42/Aβ40, P‐tau181, ApoE4	^81^
		Ig‐like domain‐containing protein, complement C1q subcomponent subunit C, complement component C9 (CO9), platelet glycoprotein Ib beta chain, Ras suppressor protein 1, disintegrin and metalloproteinase domain 10	^84^
		Plasma actins, mannan‐binding lectin serine protease 1, serum amyloid A2, fibronectin 1 (FN1), extracellular matrix protein 1, Keratin 9	^85^
Serum‐based proteins		IL‐7, TNF‐α, IL‐5, IL‐6, CRP, IL‐10, tenascin C, ICAM1	^86^
		Amino acid sequence of L/IAENR, phosphatidylcholine with two fatty‐acyl side chains, peroxidated phosphatidylcholine, oxidized glycerophosphatidylcholine	^93^
		IL‐18, I‐309	^88^
		ECH1, NHLRC2, HOXB7, FN1, ERBB2, SLC6A13	^105^
		PCSK9, F13A1, DCD coagulation factor XIII	^94^
		IL‐13, CCL17, eotaxin‐1/CCL11, CCL13, CCL4, IL‐10, CXCL10, CXCL11, IL‐12/IL‐23p40 CX3CL1, CCL8, M‐CSF, HGF, D40L, IL‐7, CCL25, IL‐2RB, IL‐15RA, CD6, VEGF‐A	^87^
		AZGP1, FBLN1, PPBP, THBS1, S100A8, S100A9	^95^
		PCK2, AK2	^21^
		Brain‐derived neurotrophic factor, insulin‐like growth factor 1, vascular endothelial growth factor, TGF‐β1, monocyte chemoattractant protein 1, IL‐18	^99^
		ITI‐H4, Cofilin 2, ApoA4, Tetranectin, AZGP1, AMBP	^90^
		IVD, CYFIP1, ADD2	^101^
		ApoC3, beta‐2‐glycoprotein 1, C4b‐binding protein alpha chain, complement C3, immunoglobulin kappa variable 2–30, alpha‐1‐antichymotrypsin isoform 1, CO9, IGHM, isoform 2, keratin, K2C6A	^96^
		CTHRC1, GFAP, OLFM3	97, 98
		Serum amyloid A4, PPBP, Platelet factor 4, ApoA4, coagulation factor X, carboxypeptidase B2, complement C1s, IGHM	^92^
		MAPT, DnaJ homolog subfamily C member 8, lysine‐specific demethylase 4D, small EDRK‐rich factor 1A, cyclin‐dependent kinase inhibitor 1, advanced glycosylation end product‐specific receptor, polycomb group protein ASXL1	^100^

### 3.1 Plasma‐based biomarker panels

Initial studies by^33^ indicated a plasma‐based panel with 18 proteins, including epidermal growth factor (EGF), interleukins (ILs), tumor necrosis factor‐alpha (TNF‐α), and monocyte colony‐stimulating factor (M‐CSF), which could differentiate subjects with AD from control cohorts without dementia with greater than 90% accuracy (*n* = 139) using a filter‐based, arrayed sandwich enzyme‐linked immunosorbent assay. This panel could also predict the progression of MCI to AD so that early intervention can be delivered to patients with presymptomatic AD.^33^ Thereafter, a plasma‐based five‐protein signature composed of IL‐1α, IL‐3, EGF, TNF‐α, and granulocyte colony‐stimulating factor, which is a subset of the 18‐protein signature described by Ray and colleagues, was identified to predict AD with 96% total accuracy (*n* = 92).^72^ However, those putative candidates were not always replicated by other researchers,^73^, ^74^ likely because different criteria were used to classify subjects with the same accuracy or because substantial preanalytical variations existed among the research cohorts. A plasma protein signature with five proteins could identify mild AD patients: apolipoprotein A‐1, α‐2‐HS‐glycoprotein, and afamin were downregulated, whereas apolipoprotein A4 (ApoA4) and fibrinogen gamma chain were upregulated in a small sample size of mild AD patients.^75^ Even though the protein signature has been validated in a small sample size of AD pathology, this protein panel should be confirmed in a large sample size for predicting AD.

To analyze the concordance among a large number of studies that used blood samples for AD biomarker discovery, Kiddle and colleagues used a proteomic Aptamer‐capture array to epitomize and reanalyze 94 of the 163 plasma protein candidates from 677 subjects included in 21 reported studies.^24^ They reidentified four candidate biomarkers—α1 antitrypsin (AAT), α‐2‐macroglobulin (A2Macro), ApoE, and complement C3—that showed a significant association with AD‐related phenotypes. Multivariate data analysis revealed that a six‐protein panel together with the subject's age as a covariate differentiated subjects with AD from control cohorts in the ADNI database with 85.4% sensitivity and 78.6% specificity.^76^ This panel consisted of AAT, A2Macro, ApoE, complement C3, and two novel biomarkers, α‐1‐macroglobulin and pancreatic polypeptide (PPP). The biomarker signature composed of AAT, A2Macro, ApoE, and complement C3 has been reidentified. PPP has been reported to show a high correlation with the brain amyloid burden,^77^ which further supports the possibility of using plasma PPP as a predictor of AD. Moreover, Park and colleagues identified five plasma biomarker candidates via mass spectrometry (MS) based‐proteomic analysis and validated these performances for AD prediction using enzyme‐linked immunosorbent assay (ELISA), A2Macro, endoplasmic reticulum aminopeptidase 2 (ERAP2), vascular endothelial cadherin 5, angiotensin‐converting enzyme, galectin3‐binding protein (LGALS3BP), which were highly predictive of brain amyloid deposition with a high accuracy of 0.871(79% sensitivity, 84% specificity) in an independent validation cohort (*n* = 254).^78^ A2Macro was once again contained in the plasma‐based protein panel identified by Park and colleagues. These consistent blood protein biomarkers suggest that the blood sample is an optimal model for the prediction of AD. However, various investigations are still needed to validate a certain plasma‐based protein panel with various proteomic data sets and various approaches to analysis.

A previous study identified 15 plasma proteins that were shown via 2‐D gel electrophoresis to differ significantly between subjects with AD and healthy elderly individuals and further validated that complement factor H and α2‐macroglobulin could be used as AD‐specific plasma biomarkers in analysis with MS immunoblot.^32^ Hye and colleagues later identified 10 plasma proteins using multiplex bead assays (Luminex xMAP), clusterin, transthyretin, cystatin C, A1AcidG, intercellular adhesion molecule 1 (ICAM1), complement C4, pigment epithelium‐derived factor (PEDF), A1AT, RANTES, apolipoprotein C3 (ApoC3), which significantly differentiated subjects with AD from those with MCI and elderly subjects without dementia (*n* = 1148) with a high degree of diagnostic accuracy (87% accuracy, 85 sensitivity, 88% specificity).^79^ ICAM1 is the only one of these proteins that overlap with the 18‐protein panel given by Ray et al. In addition, a longitudinal study of 139 patients revealed high levels of clusterin, a known amyloid chaperone, in the plasma of patients with AD.^80^ Clusterin could be remarkably expressed in specific brain regions prone to the pathophysiological processes of AD. Similarly, Kiddle et al.^24^ demonstrated that clusterin not only increased the amyloid burden but also accelerated atrophy of the entorhinal cortex and expedited the clinical pathological process in patients with AD. These results suggest that clusterin could be a promising biomarker candidate for the detection of AD.

Recently, Palmqvist and colleagues demonstrated an accurate fully automated panel of plasma biomarkers for Aβ pathology and AD dementia.^81^ They found that the combination of Aβ42/Aβ40, P‐tau181, and ApoE4 had higher accuracy (AUCs, 0.90–0.93) compared with Aβ42/Aβ40 (AUCs, 0.83–0.87) in two independent cohorts. A plasma‐based protein signature with six proteins identified AD dementia with 95% accuracy (AUC = 0.94, 95% CI = 0.87–0.98) and distinguished prodromal AD (AUC = 0.78, 95% CI = 0.68–0.87) in a total of 872 participants.^23^ Among the six proteins, oncostatin M, matrix metalloproteinase 9, hydroxyacylglutathione hydrolase, and OX‐2 membrane glycoprotein were increased, and axin‐1 and urokinase‐type plasminogen activator were decreased in Aβ‐positive individuals. Another study detailed a multianalyte classifier of 10 plasma‐based proteins, including ApoE ξ4 count, Aβ precursor protein (APP), neurofilament light (NfL) polypeptides, Aβ precursor protein‐binding family B member 3, axonemal dynein heavy chain 10, forkhead‐associated domain‐containing protein 1, neurogenin‐2, G protein–signaling modulator 2, and RE1‐silencing transcription factor and prothrombin, which could replicate the prediction of Aβ‐positive participants with a high accuracy (AUC = 0.904 specificity = 0.80, sensitivity = 0.81) using an identical proteomic workflow in an independent cohort.^82^ Moreover, this classifier to predict Aβ burden at the preclinical stage showed very similar diagnostic performance (AUC = 0.891, specificity = 0.77, sensitivity = 0.78). However, this study did not replicate Ashton's previous finding that fibrinogen γ‐chain, a protein involved in the intrinsic coagulation cascade and a target of prothrombin, predicted the neocortical amyloid burden.^83^ A recent study quantified 328 proteins in plasma exosomes and found that a diagnostic panel with 6 proteins had the capacity to differentiate AD patients from healthy controls with high accuracy.^84^ Among the six proteins, Ig‐like domain‐containing protein, complement C1q subcomponent subunit C, complement component C9 (CO9), platelet glycoprotein Ib beta chain, and Ras suppressor protein 1 were upregulated, and disintegrin and metalloproteinase domain 10 was downregulated in AD patients. Moreover, proteomic analysis showed that some proteins could be plasma‐based candidates for being AD biomarkers, such as plasma actins, mannan‐binding lectin serine protease 1, serum amyloid A2, fibronectin 1 (FN1), extracellular matrix protein 1, and Keratin 9.^85^

Based on the above, molecules containing A2Macro, α1 antitrypsin, ApoE, AAT, complement C3, clusterin, and TNF‐α were able to be duplicated in different experiments and seemed to be as a novel plasma‐based protein panel for AD prediction. It still needs to be proven whether this novel protein panel might predict AD processes. Although much work will be needed using various technologies and assays, plasma‐based signatures could be effective in predicting AD via innovative detection techniques.

### 3.2 Serum‐based biomarker panels

The exact concentrations of proteins in serum, which could differentiate patients with AD from control cohorts, is also a routine clinical practice for the prediction of AD. O'Bryant et al.^86^ duplicated a serum‐based biomarker panel with eight proteins—IL‐7, TNF‐α, IL‐5, IL‐6, IL‐10, ICAM1, tenascin C, and C‐reactive protein (CRP)—that had 95% accuracy (88% sensitivity, 92% specificity) for the detection of AD in a cohort with 300 subjects. Notably, the accuracy significantly improved up to 98% by the inclusion of demographic factors, such as age, sex, education, and APOE4 status. Villarreal and colleagues identified two serum‐based proteins, IL‐18 and T‐lymphocyte‐secreted protein (TLSP), which differentiated subjects with AD from control subjects with AUC of 0.94 (86% sensitivity, 90% specificity) in a cohort with 135 participants: the level of IL‐18 was significantly elevated but TLSP was deceased in the former.^88^ Moreover, Yu et al.^89^ have demonstrated an eight protein‐based biomarker panel that could distinguish AD from control candidates with a sensitivity of 97.7%, specificity of 88.6%, and AUC of 99%. The results showed that serum leptin, IL‐1α, IL‐3, PAI‐1, and TNF‐α levels, had a strong positive correlation and were upregulated in AD patients, while CXCL10, IL‐13, and resistin displayed a significant inverse correlation. These studies indicated that an AD blood signature is probably associated with inflammatory risk factors. A serum‐based panel with six proteins—inter‐alpha‐trypsin inhibitor heavy chain H4 (ITI‐H4), ApoA4, Cofilin 2, Tetranectin, Zinc‐alpha‐2‐glycoprotein (AZGP1), and alpha‐1‐microglobulin/bikunin precursor (AMBP)—were altered that was identified in AD patients.^90^ In this research, the authors demonstrated that the full size of the ITI‐H4 protein was increased, while a fragment of ITI‐H4 was decreased in AD patients, and Cofilin 2, ApoA4, Tetranectin, AZGP1, and AMBP were significantly increased in AD patients. Sun and colleagues have also shown that Cofilin 2 was increased significantly in the serum of AD patients.^91^

A novel serum‐based protein profile with eight proteins—serum amyloid A4, pro‐platelet basic protein (PPBP), platelet factor 4, ApoA4, coagulation factor X, carboxypeptidase B2, complement C1s, and immunoglobulin heavy constant mu (IGHM)—was found and could identify AD case from controls with AUC of 92.3%.^92^ A serum‐based biomarker panel with four proteins, including an amino acid sequence of L/IAENR, phosphatidylcholine with two fatty‐acyl side chains, peroxidated phosphatidylcholine, and oxidized glycerophosphatidylcholine, was identified using multiple assays for the prediction of AD.^93^ Another serum‐based biomarker signature with pro‐protein convertase subtilisin/kexin type 9 (PCSK9), coagulation factor XIII, A1 polypeptide (F13A1), and dermcidin (DCD) was identified using liquid chromatography–tandem MS (LC–MS/MS) for the prediction of AD, followed by validation by enzyme‐linked immunosorbent assay and western blotting.^94^ Consistently, LC–MS/MS coupled with PiB‐PET imaging revealed that three of the candidates were altered significantly in AD brain tissues, which suggests that pro‐protein convertase PCSK9, F13A1, and DCD could be potential candidate biomarkers for early diagnosis of AD.

Shen et al.^95^ detected differential expressions of AZGP1, fibulin‐1, PPBP, thrombospondin‐1, S100 calcium‐binding protein A8, and S100 calcium‐binding protein A9 in the sera of subjects with AD compared with control subjects using the proteomics approach of isobaric tagging for relative and absolute quantitation (iTRAQ), which indicates that the serum‐based biomarker panel with six proteins might have potential for the detection of AD. Soares Martins and colleagues reported a serum‐based proteomic signature with nine exosome proteins that could identify AD cases from controls using MS.^96^ They found that ApoC3, beta‐2‐glycoprotein 1, C4b‐binding protein alpha chain, complement C3, and immunoglobulin kappa variable 2–30 were significantly increased in controls, whereas alpha‐1‐antichymotrypsin isoform 1, CO9, IGHM, isoform 2 and keratin, type II cytoskeletal 6A (K2C6A) were significantly increased in AD cases. Consistently, upregulated CO9 and downregulated ApoC3 were in both serum and plasma of AD patients.^84^, ^96^ Dey's group^21^ recently analyzed 4826 serum protein components, covering at least six orders of magnitude in dynamic range, and defined the variability in AD and control groups using tandem mass tag LC/LC–MS/MS platform. Proteomic analysis showed that 30 proteins could significantly differentiate subjects with AD from control subjects, including 26 with decreased expression and 4 with increased expression. Importantly, these proteins are associated with the mitochondrial pathway, fatty acid beta‐oxidation, and AGE/RAGE. They further subjected the 30 target proteins to a multiplexed targeted LC–MS method (TOMAHAQ) and confirmed phosphoenolpyruvate and adenylate kinase 2, mitochondria‐related proteins, as novel biomarker candidates for the prediction of AD. The research group subsequently revealed an AD‐correlated protein panel of CTHRC1, GFAP, and OLFM3 across cortex and serum by integrating all proteomic data sets.^97^, ^98^ Notably, 59% of the protein signatures across cortex, CSF, and serum were mitochondria proteins, suggesting mitochondrial dysfunction in AD. A serum‐based panel with six proteins, including brain‐derived neurotrophic factor, insulin‐like growth factor 1, vascular endothelial growth factor, transforming growth factor‐beta type 1 (TGF‐β1), monocyte chemoattractant protein 1, and IL‐18, could differentiate subjects with AD from the detected cohorts with great accuracy (AUC = 0.85).^99^

A novel serum signature with seven autoantibody candidates, including microtubule‐associated protein tau (MAPT), DnaJ homolog subfamily C member 8, lysine‐specific demethylase 4D, small EDRK‐rich factor 1A, cyclin‐dependent kinase inhibitor 1, advanced glycosylation end product‐specific receptor, and polycomb group protein ASXL1, could identify AD cases from controls with high accuracy (AUC = 0.94) in four cohorts.^100^ Importantly, these autoantibodies could distinguish AD from other neurodegenerative diseases and outperformed amyloid and tau protein concentrations in cerebrospinal fluid in predicting cognitive decline (*p* < 0.001). This research implied that systemic immune signals might originate outside the brain against AD. Previously, San Segundo‐Acosta et al.^101^ also found another serum protein‐epitope panel, including isovaleryl‐CoA dehydrogenase (IVD), cytoplasmic FMRI‐interacting protein 1 (CYFIP1), and beta adducing protein 2 (ADD2), could distinguish AD patients from controls by comprehensive multiomics approaches. IVD was significantly two‐fold higher in AD patients than in controls, whereas CYFIP1 and ADD2 were downregulated in the prefrontal cortex of AD. Taken together, although numerous studies have identified and demonstrated distinct blood‐based biomarker panels, these panels almost certainly need to be refined, simplified, and validated in independent cohorts.

### 3.3 Discovery of biomarkers by crossing serum and plasma for prediction of AD

Because the pathophysiological mechanism of AD is complex, the application of serum‐ or plasma‐based protein biomarkers alone seems inadequate for practical prognosis or diagnosis of AD. Several studies have observed some differences in the proteomic profiles of plasma and serum,^102^ which may be the reason why blood protein biomarkers are difficult to duplicate for the prediction of AD. A consistent series of proteins identified from serum and plasma via multiple analysis approaches could be considered a promising biomarker for AD prediction. However, these requirements improve the difficulty and complexity of identification. Currently, only a few studies have reported several consistent biomarkers identified in both the serum and plasma of subjects with AD (Table 2).

**TABLE 2:** Overview of protein biomarkers in both serum and plasma for diagnosis of Alzheimer's disease.

Identified protein biomarkers	Changes	Reference
TNF‐α	**↑**	33, 72, 86
NfL	**↑**	13, 33, 82, 103
ICAM1	**↑**	33, 79, 86
M‐CSF	**↑**	33, 87
CRP	**↓**	86, 104
Factor VII, MCP	**↓**	^104^
PPP	**↑**	76, 77, 104
Adiponectin, tenascin C, VCAM1, β2M, FABP	**↑**	86, 104
ApoC3	**↓**	84, 96
CO9	**↑**	84, 96
Complement C3	**↓**	24, 76, 96
IL‐18	**↑**	88, 99, 104
ApoA4	**↑**	75, 90, 104
TLSP	**↓**	88, 104

Previous studies have shown that a candidate biomarker neurofilament light (NfL) protein is increased in the plasma and CSF of patients with AD.^33^, ^82^ Serum/plasma NfL expression is also significantly elevated in patients with AD.^103^ Serum neurofilament dynamics monitored neurodegeneration and clinical progression in AD.^13^ Furthermore, O'Bryant and colleagues found that TNF‐α and ICAM1, which were initially involved in the 18 plasma‐based proteins of Ray et al., could be biomarker candidates for the prediction of AD, as they were significantly elevated in the serum of AD subjects.^86^ Increased levels of M‐CSF have also been shown in the plasma^33^ and serum Brunner et al.^87^ of subjects with AD. CO9 was upregulated, and ApoC3 was downregulated in both serum and plasma of AD patients.^84^, ^96^ In addition, O'Bryant et al.^104^ cross‐validated 11‐protein serum‐plasma risk score with a specific algorithm and found that the expressions of CRP, factor VII, and monocyte chemotactic protein (MCP) were decreased in the body fluids of patients with AD (serum, plasma, and CSF). Adiponectin, tenascin C, PPP, vascular cell adhesion molecule 1 (VCAM1), and beta 2 microglobulin (β2M) were increased in various fluids, and fatty acid binding protein (FABP), ApoA4, and IL‐18 were increased in serum and plasma, as opposed to TLSP. An increased plasma PPP has been reported to have a high correlation with the brain amyloid burden.^77^ Notably, Long et al.^105^ used serum samples and RNA profile samples from subjects with AD to identify biomarkers for AD diagnosis and discovered a novel biomarker set that includes enoyl‐coenzyme A hydratase 1, NHL repeat containing 2, homeobox B7, FN1, neuro/glioblastoma‐derived oncogene homolog (avian) transcript variant 2, and solute carrier family 6 (neurotransmitter transporter, GABA), member 13 with high sensitivity and accuracy. Complement C3 was indicated to be reduced in both serum and plasma of AD cohorts (^24^, ^76^, ^96^;). Lista et al. previously reviewed serum‐ or plasma‐based proteomic biomarkers from subjects with AD for the prediction of AD.^106^ Zürbig and Jahn summarized the protein changes in the body fluids of AD patients analyzed with proteomic techniques and identified 366 proteins and peptides that could be targets for AD.^107^

Taken together, we conclude that some consistent biomarkers, such as TNF‐α, NfL, ICAM1, M‐CSF, CRP, factor VII, MCP, adiponectin, tenascin C, PPP, FABP, TLSP, ApoA4, CO9, ApoC3, VCAM1, complement C3, and β2M, have been identified in both the serum and plasma of AD cohorts and could serve as reliable candidate biomarkers for the prediction of AD. However, for these potential biomarkers to be used in the clinic, the assay development and validation must be completed. Furthermore, the transition of the discovered biomarkers from a research setting to a clinical setting would be ensured to be consistent at the level of individual patients, and the discovered biomarkers would be stable under prevalent conditions. In addition, a substantial improvement in proteomic exploration of blood‐based biomarkers for AD prediction would be made possible by innovations in analytical instrumentation, particularly in the innovative MS arena.

## 4 BLOOD‐DERIVED BIOMARKERS ANALYZED BY METABOLOMICS FOR AD PREDICTION

Metabolomics, often called global metabolic profiling, is the systematic profiling of metabolites; it can be used to identify dynamic, qualitative, and quantitative alterations of several metabolites in a biological system.^108^ In terms of metabolites, lipids, choline, carnitine amino acids, and nicotinamide derivatives and other metabolites (depsides, tocopherols, dipeptides, Lyso PEs, inositol derivatives) participate in life activities and are involved in lipid metabolism, the energy metabolism, the amino acid metabolism, and the cholinergic system. Because of its potential, metabolomics has been increasingly applied in various biofluids, especially blood, to differentiate subjects with AD from control subjects (^109^, ^110^). Metabolomic approaches that are routinely used in the detection of AD biomarkers contain nontargeted and targeted metabolomics; that is, differentially expressed metabolites are identified by nontargeted metabolomics, followed by targeted metabolomics to quantify the accuracy, sensitivity, and specificity of potential biomarkers in blood. Recent studies have used metabolomics to reveal that some metabolic pathways are altered in the blood of subjects with AD, such as the lipid metabolism including sphingolipid, phospholipid, and cholesterol; the amino acid metabolism; the energy metabolism; the tricarboxylic acid (TCA) cycle; neurotransmission and inflammation^111^; and abnormal metabolite levels (tyrosine, glycylglycine, glutamine, lysophosphatic acid, platelet‐activating factor, organic acids, isoprostanes, and prostaglandins).^112^ For instance, of the 249 metabolites in blood of 5274 participants with dementia analyzed, 130 were significantly associated with AD, and among metabolites, lipoprotein lipid, linoleic acid, sphingomyelin, glucose, and branched‐chain amino acids ranked top in importance.^113^ Table 3 summarizes blood‐based metabolites that could provide an indication for AD prediction.

**TABLE 3:** Blood‐based candidate biomarkers analyzed by metabolomics for Alzheimer's disease prediction.

Metabolic pathways	Candidate biomarkers	Changes	References
Phospholipid metabolism	PC aa C36:6, PC aa C38:0, PC aa C38:6, PC aa C40:1, PC aa C40:2, PC aa C40:6, PC ae C40:6, lysoPC a C18:2	↓	^115^
	LysoPC/PC ratio	↓	119, 120
	PC aa C36:5, PC aa C38:6, PC aa C40:6, PC16:0/20:5, PC16:0/22:6, PC18:0/22:6	↓	^116^
	PC aa C32:0, PC aa C34:0, PC aa C34:4, PC ae C36:4, PC aa C38:3, PC aa C40:5, PC ae C42:1	↓	^125^
	Lysophosphatic acid (C18:2)	↓	^112^
	PC aa C40: 6, PC ae C40: 1	↓	^123^
	Glycerophosphocholine	↓	^117^
	PE	↓	^124^
	Lyso‐PE	↑	
	PC‐DHA	↑	^118^
	Lyso PC (18:1)	↑	121, 122
Sphingolipid metabolism	SM C16:0, SM C16:1, SM C18:1, SM (OH) C14:1	↑	^123^
	Dihydro SM, SM/Cer ratios	↑	^130^
	SM d18:1/20:1	↑	^112^
	Cer d18:1/16:0, HexCer d18:1/18:0	↑	^128^
	SM d16:1/22:0, GM3 d16:1/22:0, GM3 C18:1/16:0, Cer d16:1/24:0	↓	
	Cer (d18:0/16:0), Cer (d19:1/24:0), Cer (d18:0/23:0), Cer (d18:2/25:0)	↑	^129^
	Cer (d18:1/23:0), Cer (t16:1/14:0), Cer (m18:1/20:0), Cer (m18:0/22:0), Cer (d19:1/22:0)	↓	
	Sphingomyelin, sphingosine	↑	^134^
	Sphinganine‐1‐phosphate, sphingosine‐1‐phosphate	↓	
	Glycosyl‐N‐stearoyl sphingosine (d18:1/18:0), glycosyl Cer d18:2/24:1	↓	^132^
	Cer C22:0, Cer C24:0	↓	^131^
Carnitine metabolism	C3, C16:1‐OH	↓	^115^
	C5, C5‐OH/C3‐DC‐M, C9, C10:2, C18:1‐OH, C10:1, C12:1, C16:2	↓	^125^
	C2, C12, C12:1, C14:1	↓	144, 145
	C8, C10:1,C10, C0, C14:2, C2, oleylcarnitine	↓	^144^
	C5, C8, C10:1, C10	↓	^146^
	C3, C5	↓	^147^
	C12, C14:1, C14:2, C16:1, C18	↑	
	C10, C8, C5‐DC/C6‐OH	↑	^148^
Amino acid metabolism	Pyruvic acid, Val	↓	^155^
	Val, Ile	↓	^122^
	Trp	↓	122, 156, 158
	Val, His, Trp, Lys, Tyr, sarcosine	↓	^147^
	Glu, Tyr, Gly, Phe	↓	^153^
	Tyr, glycylglycine, and glutamine	↓	^112^
	Asn, asymmetric dimethylarginine	↓	^125^
	Asn, Met, His, acetyl‐spermidine	↓	^146^
	D‐Ser, D‐Asp, D‐Ala, D‐Leu, D‐Pro	↓	^152^
	D‐Phe	↑	
	GABA, 4‐aminobutanal, L‐ornithine	↓	^160^
	Creatine, spermine, N‐acetylputrescine, N1, N12‐diacetlyspermine	↑	
	Glutamine	↓	^136^
		↑	^154^
	Gly, Pro	↑	^148^
Energy metabolism	Glyceric acid, fructose, glucosaminic acid, succinic acid, glutamic acid	↓	^164^
	Piperine	↓	^154^
	Halogenated compounds	↑	
	Uridine	↓	^163^
	Glycolysis, lactate	↑	
	Citric acid	↑	^162^
	O‐Acetyl‐glycoproteins	↑	^153^
Choline system	Choline	↑	^165^
	Choline transmitter	↓	
Alternation of cholesterol metabolism	Total cholesterol	↑	^135^
	Low‐density lipoprotein cholesterol	↑	^136^
	24S‐hydroxycholesterol	↑	^137^
		↓	^139^
Oxidative stress pathway	AFMK	↑	167, 173
	Pyrogallol‐2‐O‐glucuronide	↑	166, 173
Melatonin metabolism	Glycoursodeoxycholic acid	↑	^115^
Others	1D‐myoinositol‐1,3,4,6‐tetrakisphosphate, D‐myo‐inositol‐3,4,5,6‐tetrakisphosphate, 1D‐myo‐inositol‐1,4,5,6‐tetrakisphosphate, inositol‐1,3,4,5‐tetraphosphate	↑	^165^
	Organic acids, isoprostanes, prostaglandins, acetate		112, 153

### 4.1 Abnormal phospholipid metabolism

Phospholipids, the principal membrane‐forming lipid family that influences many cell processes, such as proliferation, trafficking, and modulation of membrane proteins and their functions, play a dominant role in AD progression.^114^ Unambiguous identification using stable isotope dilution‐MRM‐MS revealed significantly lower levels of phosphatidylcholines in the plasma of participants with AD with greater than 90% accuracy.^115^ They found that the levels of phosphatidylcholines [PCs; PC diacyl (aa) C36:6, PC aa C38:0, PC aa C38:6, PC aa C40:1, PC aa C40:2, PC aa C40:6, PC acyl‐alkyl (ae) C40:6] and lysophophatidylcholine (lysoPC a C18:2) were significantly reduced in the plasma.^115^ In line with these findings, lower plasma levels of PC aa C36:5, PC aa C38:6, PC aa C40:6, and phosphatidylcholines (PC16:0/20:5, PC16:0/22:6, PC18:0/22:6) were closely associated with the pathogenesis of AD.^116^ Decreased glycerophosphocholine in plasma could identified AD patients from controls and was as a dementia biomarker.^117^ Abnormal levels of lysophosphatic acid (C18:2) were also indicated in AD.^112^ Furthermore, higher levels of phosphatidylcholine (PC) docosahexaenoic acid (PC‐DHA) in plasma also increase the risk of developing AD.^118^ A decreased ratio of lysophosphatidylcholines to phosphatidylcholines (lysoPC/PC) in plasma has been shown to differentiate patients with AD from control subjects.^119^ Previous evidence showed that lysoPC/PC ratios were also decreased in the CSF of patients with AD.^120^ In addition, Lyso PC (18:1) was confirmed to differentiate early AD patients from healthy control participants, specially AD patients with *ApoE ε4* gene,^121^ which was consistent with a study.^122^ Levels of Lyso PC (18:1) in plasma were significantly increased in patients with AD. Interestingly, Chang et al.^122^ showed that AD was associated with the altered metabolism of PCs in the serum of females.

Another indicator of the progression of AD is an alteration in the levels of glycerophospholipids in the blood. For instance, lower levels of glycerophospholipids (PC aa C40: 6 and PC ae C40: 1) in the blood are involved in the progression of AD, leading to decreases in attention and impairment of language function.^123^ In addition, low levels of serum phosphatidylethanolamine (PE) and high levels of lysophosphatidylethanolamine (lyso‐PE) were significantly associated with increased risk of AD and led to twofold faster median time to progression from MCI to AD with hazard ratios 0.62 and 1.34, respectively.^124^ This research suggests that PE and lyso‐PE might be biomarkersfor predicting conversion from MCI to AD. Fiandaca and colleagues found that levels of glycerophosphatidylcholines (PC aa C32:0, PC aa C34:0, PC aa C34:4, PC ae C36:4, PC aa C38:3, PC aa C40:5, and PC ae C42:1) were significantly decreased in subjects with AD relative to control subjects.^125^ Although the results remain inconsistent, the significant changes in phospholipid metabolites in the blood of patients with AD suggest an indicator for preclinical detection. Correcting the disorder in the phospholipid metabolism could become a powerful solution to reverse AD pathology.

### 4.2 Abnormal sphingolipid metabolism

Sphingolipids consist of multiple species including sphingomyelins, ceramides, and sphingosines which can be interconverted by a characterized metabolic pathway. Because sphingolipids are enriched in myelin and are also closely associated with lipid rafts, abnormal metabolism of sphingolipids could be involved in inflammation and amyloidogenesis‐related neuron death, and in the pathogenesis of AD. Indeed, sphingolipids are related to tau protein hyperphosphorylation, Aβ metabolism, calcium homeostasis, and acetylcholine biosynthesis.^123^ Most of sphingolipids were downregulated in AD patients.^126^, ^127^ However, some sphingolipids have been shown high levels in blood of patients with AD.^128^, ^129^ Abnormal metabolites of sphingolipids could be as a blood‐based biomarker for AD prediction, and compounds that could correct sphingolipid metabolism might show some benefits for AD patients. For instance, Mielke and colleagues found that sphingomyelin (SM)/ceramides (Cer) ratios and dihydro SM were increased in the blood of AD patients.^130^ Varma et al.^123^ identified a serum‐based biomarker panel with four distinct sphingolipid species—SM with acyl residue sums C16:0, C18:1, and C16:1 (SM C16:0, SM C16:1, SM C18:1) and hydroxyl SM with acyl residue sum C14:1 (SM (OH) C14:1)—that could discriminate between AD subjects and ADNI controls and were consistently associated with severity of AD pathology at autopsy and AD progression across prodromal and preclinical stages. Higher log‐transformed blood concentration of four sphingolipids in normal individuals were significantly associated with increased risk of AD: SM C16:0 (hazard ratio [HR] = 4.430, *p* = 0.002), SM C16:1 (HR = 3.455, *p* = 0.003), SM C18:1 (HR = 2.255, *p* = 0.038), and SM (OH) C14:1 (HR = 3.539, *p* = 0.009). Similarly, SM d18:1/20:1 was overexpressed in plasma of patients with AD.^112^ These research suggest that some certain increased sphingolipid species, such as most of sphingomyelins, could be for biomarkers of monitoring AD.

Chua et al.^128^ identified a panel of sphingolipids—SM d16:1/22:0, GM3 d16:1/22:0, GM3 d18:1/16:0, ceramides (Cer) d16:1/24:0, Cer d18:1/16:0, cerebrosides (HexCer) d18:1/18:0—that could classify patient with AD from controls with a high accuracy (AUC = 0.812) by performed regression with Least Absolute Shrinkage and Selection Operator. In the study, the authors demonstrated that Cer d18:1/16:0 and HexCer d18:1/18:0 were upregulated, and Cer d16:1/24:0, GM3 d16:1/22:0, GM3 d18:1/16:0, and SM d16:1/22:0 were downregulated in AD cases. Liu and colleagues also confirmed that different lipid profiles could discriminate AD patients from controls.^129^ They found that Cer (d18:1/23:0), Cer (t16:1/14:0), Cer (m18:1/20:0), Cer (m18:0/22:0), and Cer (d19:1/22:0) were deceased in patients with AD, whereas Cer (d18:0/16:0), Cer (d19:1/24:0), Cer (d18:0/23:0), and Cer (d18:2/25:0) were increased in patients with AD, which were positively correlated with AD. Another study of the association between ceramides with cognitive decline and hippocampal volume loss showed that patients with MCI displayed significantly lower plasma levels of ceramide Cer C22:0 and Cer C24:0 (0.94–0.96‐fold) than control subjects.^131^ Within the MCI cohorts, a higher baseline of Cer C22:0 and Cer C24:0 in blood could predict hippocampal volume loss and cognitive impairment over the course of 1 year,^131^ thus making it a potential early indicator of AD progression. These studies indicate that abnormal sphingolipid metabolism involved altered ceramides and sphingosines in blood of AD cases.

Moreover, the erythrocyte metabolites could identify AD cases from controls. Mill et al. found that metabolites associated with lipid metabolism in particular sphingolipid species glycosyl‐N‐stearoyl‐sphingosine (d18:1/18:0) and glycosyl Cer (d18:2/24:1) were significantly decreased in erythrocytes of AD patients.^132^ Dehghan and colleagues^133^ showed that plasma lactosylceramides concentrations were associated with AD risk using ultra‐performance liquid chromatography–mass spectrometry (UPLC–MS). Moreover, Lin and colleagues demonstrated typical biomarkers of sphingolipid intermediates, including decreased sphinganine‐1‐phosphate and sphingosine‐1‐phosphate, increased sphingomyelin and sphingosine, in the plasma of subjects with AD with a high accuracy (AUC = 1.0, 1.0, 0.82, 0.68, respectively) using ultra‐performance LC–quadrupole time‐of‐flight MS and ultrahigh‐performance LC‐Q‐Exactive‐MS.^134^

From above, the abnormal levels of blood sphingolipid metabolites have been indicated to be associated with disease progression during prodromal and preclinical stages of AD. Although sphingolipids might be relevant biomarkers for the early detection of AD, a large sample of extremely sensitive clinically relevant tests would be required to detect the changes in sphingolipid metabolites in the blood in relation to baseline levels and verify sphingolipid metabolite–specific biomarkers to predict AD. In addition, a standard protocol would need to be established to obtain and process the blood samples.

### 4.3 Abnormal cholesterol metabolism

The balance of cholesterol metabolism is crucial for maintaining the activities of cells and organisms. Cholesterol in the pathogenesis of AD is a genetic variant of *ApoE*, and high levels of ApoE in plasma are associated with abnormal cholesterol metabolism, and a high level of total cholesterol in serum during midlife confers an increased risk of AD,^135^ which suggests intrinsic alterations of the blood‐based cholesterol metabolism in AD. Consistent with previous studies, high levels of low density lipoprotein cholesterol increased the risk of AD.^136^ These researches suggest that abnormal cholesterol metabolism in the blood might predict AD procession. Although high levels of cholesterol are indicated in AD processes, some studies showed conflicting results on cholesterol metabolites (24S‐hydroxycholesterol) in the blood of patients with AD. An independent experiment reported a significant increase in 24S‐hydroxycholesterol levels in the plasma of patients with late‐onset AD.^137^ In contrast, several articles demonstrated reduced levels of plasma 24S‐hydroxycholesterol in subjects of AD and MCI.^138^, ^139^ This contrasting role of plasma 24S‐hydroxycholesterol could be due to heterogeneity regarding the different stages of AD. Moreover, it has been reported that higher levels of plasma 24S‐hydroxycholesterol were also likely to develop incident cognitive impairment.^140^ However, the ratio of 24S‐hydroxycholesterol to total circulating cholesterol was found to be significantly decreased in AD and MCI compared to controls.^141^ These results suggest that 24S‐hydroxycholesterol corrected by plasma cholesterol levels could be more informative for a biomarker candidate for the diagnosis of AD.

### 4.4 Abnormal carnitine metabolism

Carnitine is an endogenous molecule involved in fatty acid metabolism that primarily facilitates the mitochondrial β‐oxidation of long‐chain fatty acids and has been proposed for treating AD.^142^, ^143^ Recent metabolomics studies of the plasma of subjects with AD have highlighted metabolic alterations in carnitine levels, and a targeted metabolomic and lipidomic analysis demonstrated that acylcarnitines (ACs; propionyl‐carnitine [C3] and C16:1‐OH) were nearly depleted in the plasma of subjects with AD relative to control subjects.^115^ A plasma metabolite panel was later identified from subjects with AD that included nine ACs: propionyl‐carnitine (C3), valeryl‐l‐carnitine (C5), hydroxyvaleryl‐l‐carnitine/methylmalonyl‐l‐carnitine (C5‐OH/C3‐DC‐M), non‐ayl‐l‐carnitine (C9), decadienyl‐l‐carnitine (C10:2), hydroxyoctadecenoyl‐l‐carnitine (C18:1‐OH), decenoyl‐l‐carnitine (C10:1), dodecenoyl‐carnitine (C12:1) and hexadecadienyl‐l‐carnitine (C16:2).^125^ All these nine metabolites were significantly decreased in the plasma of patients with AD. Moreover, two independent studies reported that acetyl‐carnitine (C2), dodecanoyl‐carnitine (C12), C12:1, and tetradecenyl‐carnitine (C14:1) were reduced in the plasma^144^ and serum^145^ of patients with AD. Furthermore, octanoylcarnitine (C8), C10:1, decanoylcarnitine (C10), carnitine (C0), tetradecadienoylcarnitine (C14:2), oleylcarnitine, and acetylcarnitine (C2) were lower in the plasma of patients with AD compared with controls.^144^ C5, C8, C10:1, and C10 were decreased in their serum of patients with AD.^146^ On the contrary, C12, C14:1, and C14:2 were shown a significant increase in the blood during the course of AD progression.^147^ Besides, they found that hexadecenoylcarnitine (C16:1) and octadecenoylcarnitine (C18:1) were also significantly increased in the course of AD progression. However, C3 and C5 were shown the significant decrease during the course of AD progression, which was consistent with previous studies.^115^, ^125^ Although some results were not consistent, these alternations suggest that abnormal fatty acid metabolism might serve as a potential biomarker for the early diagnosis of AD.

Notably, Arnold et al.^148^ investigated the effects of sex and the *APOE* ξ4 genotype on metabolic alterations related to representative A‐T‐N biomarker profiles by analyzing 139 blood metabolites from 1517 participants of the ADNI cohorts (A: CSF Aβ1–42 pathology; T: CSF p‐tau; N: region of interest‐based glucose uptake measured by [18F] fluorodeoxyglucose‐PET). They found a significant female‐specific association shown by higher levels of acylcarnitine C10 with increased CSF p‐tau, and two metabolites (C8 and C5‐DC/C6‐OH) of this pathway fell short of meeting the Bonferroni threshold. This indicates that aggregations of medium‐chain fatty acids are a female‐specific risk factor for AD, which suggests high energy demands coupled with mitochondrial beta oxidation‐impaired energy production.^149^ The accumulated evidence demonstrates that carnitine and related metabolites could serve as blood‐based biomarkers for the prediction of AD. However, many inconsistencies remain; for example, most carnitine and related metabolites have not been identified in both serum and plasma.

### 4.5 The alternation of amino acid metabolism

Various metabolic pathways were affected in AD cases, such as the arginine, alanine, aspartate, glutamate, and pyruvate metabolism pathways,^85^ suggesting that some amino acids and their metabolites were altered during AD progression. A previous study has shown that some certain amino acids or metabolites, including asparagine (Asn), methionine (Met), histidine (His), and acetyl‐spermidine, were significantly reduced in these serum samples of patients with AD.^146^ Abnormal levels of D‐amino acids have been implicated in the pathogenesis of AD.^150^, ^151^ A study using ultra‐performance LC–MS/MS analysis revealed that the concentrations of D‐serine (D‐Ser), D‐aspartate (D‐Asp), D‐alanine (D‐Ala), D‐leucine (D‐Leu), and D‐proline (D‐Pro) were significantly lower in the plasma of subjects with AD than that of the normal control subjects, whereas the content of D‐phenylalanine (D‐Phe) was higher.^152^ A multiscale metabolite co‐expression network analysis of the blood metabolites showed that valine (Val), histidine (His), tryptophan (Trp), lysine (Lys), tyrosine (Tyr), and sarcosine were significantly decreased during the course of AD progression, which have been confirmed in the MCI and AD groups (*p* < 0.05).^147^ Lower serum levels of glutamate (Glu), tyrosine (Tyr), glycine (Gly), and phenylalanine (Phe) were also associated with a significantly greater risk of developing dementia.^153^ Moreover, abnormal metabolite levels of tyrosine (Tyr), glycylglycine, and glutamine in the plasma were also implicated in AD processes.^112^ These studies indicate that amino acids and their metabolites could be blood‐based biomarkers for AD prediction or diagnosis.

A metabolomics study also identified an elevated level of glutamine in the plasma of subjects with AD dementia using metabolome‐wide association study meta‐analysis.^153^, ^154^ In contrast, low levels of glutamine were associated with an increased risk of AD using Mendelian Randomization Robust Adjusted Profile Score analysis.^136^ These results show that different analysis methods and sample sizes could display different results. Therefore, it is necessary to unify a single analysis method and increase the sample size in order to find accurate metabolite biomarkers that could predict AD. A recent study showed that a panel of serum‐derived metabolites—pyruvic acid and valine (Val)—were significantly altered in AD patients using sparse‐partial least squares discriminant analysis.^155^ Pyruvic acid and Val were consistently reduced in AD patients, and pathway analysis revealed that they were involved in branched‐chain amino acids (BCAAs) and energy metabolism. Moreover, levels of tryptophan (Trp) were decreased in blood of AD patients using meta‐analysis,^156^ which was positively correlated with the corresponding CSF level.^157^ The decrease of tryptophan in AD patients is likely because the significantly upregulated cytokines and inflammatory factors in AD patients increase the conversion of tryptophan to canine uric acid. A metabolite, indole‐3‐propionic acid, an indole derivative converted from tryptophan by gut bacteria, was identified in AD progression from controls.^158^ This research suggests that tryptophan and its downstream metabolites could be used for AD diagnosis. Shao and colleagues identified a metabolite signature with five metabolites, comprising tryptophan, cholic acid, chenodeoxycholic acid, allocholic acid, and indolelactic acid, which were able to distinguish patients with AD from controls with satisfactory sensitivity and specificity.^159^ Higher levels of glycoursodeoxycholic acid were found in the subjects with MCI/AD than in the control subjects.^115^ These indicate tryptophan might play a dominant role in blood‐based biomarkers for AD prediction. Interestingly, a study showed that AD was mainly associated with changes in amino acid in males and tryptophan in females.^122^ They also found that BCAAs (Val) and isoleucine (Ile) were decreased in *APOE ε4+* AD cases compared to controls, suggesting that metabolic changes that could distinguish AD patients from controls were able to be affected by sex and *APOE*. The significant male‐heterogeneous association presented in this study is that a higher level of glycine is linked to an increased level of CSF p‐tau,^148^ which also indicates the upregulation of energy demands. Importantly, identification by twofold stratification of *APOE* ξ4 and sex showed that a higher level of proline (Pro) is the greatest risk factor for AD, particularly in *APOE* ξ4+ females.^148^ In addition, a plasma metabolite panel that could predict preclinical transition to clinical stages of AD includes decreased levels of asparagine (Asn) and asymmetric dimethylarginine.^125^ Graham and colleagues analyzed the plasma metabolome from 700 individuals in the ADNI cohort using a variety of contrasts and revealed disruption of the polyamine and l‐arginine (L‐Arg) metabolism during early AD processes.^160^ In that study, they showed that the concentrations of GABA, 4‐aminobutanal, and L‐ornithine decreased in subjects with AD and that the concentrations of N1, N12‐diacetlyspermine, creatine, spermine, and N‐acetylputrescine increased in each stage from MCI to AD stages. In the late stages of AD, a shift to amino acids occurs, with a specific perturbation of the arginine metabolism.^161^ Metabolites in plasma, including quinolinic acid, kynurenine, and indoxyl‐sulfate, were increased in dementia patients compared with healthy elderly subjects and differed significantly.^117^ These data indicate that dysregulation of the peripheral amino acid metabolism may be a common feature of AD.

### 4.6 Abnormal metabolites related to energy metabolism

Disorders of core energy metabolism are also indicated by significant enrichment, including decreased piperines and elevated halogenated compounds, in the plasma of subjects with AD.^154^ After absolute quantification, the concentration of intermediates from the TCA cycle in plasma and CSF increased significantly, such as citric acid in patients with AD.^162^ A study of 1440 individuals using an NMR platform demonstrated that some metabolites involved in energy metabolism showed a significant association with incident AD, including low levels of glutamate and high levels of O‐acetyl‐glycoproteins.^153^ In addition, evidence has shown alteration of plasma metabolites related to the cellular energy metabolism in patients with AD, such as high levels of glycolysis and lactate.^163^ They also observed significant decreases in the levels of uridine in subjects with AD. Recently, untargeted metabolomics analysis showed lower levels of glyceric acid, fructose and glucosaminic acid, succinic acid, and glutamic acid in the blood of AD subjects,^164^ further suggesting abnormal energy metabolism of AD patients.

Taken together, this evidence indicates that metabolites related to energy metabolism might be also potential biomarkers for the prediction of AD, but further investigation would be needed for verification.

### 4.7 Other metabolites

In addition to the fatty acid/lipid metabolism, energy metabolism, and traditional metabolites, Peña‐Bautista et al.^165^ showed that the increased plasma‐based choline level in subjects with MCI‐AD was the result of a compensatory reaction caused by a decrease in cholinergic transmission, which suggests reliable detection for early AD diagnosis. They also revealed that the metabolites of the inositol pathway were downregulated in subjects with MCI‐AD, including 1D‐myo‐inositol‐1,4,5,6‐tetrakisphosphate, 1D‐myoinositol‐1,3,4,6‐tetrakisphosphate, D‐myo‐inositol‐3,4,5,6‐tetrakisphosphate, and inositol‐1,3,4,5‐tetraphosphate.^165^ Habartová et al. (2019) identified two molecules, increased pyrogallol‐2‐O‐glucuronide and increased acetyl‐N‐formyl‐5‐methoxykynurenamine (AFMK), which are believed to be related to AD. Pyrogallol‐2‐O‐glucuronide has been reported to bind oxygen.^166^ The increase in pyrogallol‐2‐O‐glucuronide in subjects with AD may thus reflect oxidative stress that leads inevitably to neuronal damage. AFMK is a by‐product of melatonin metabolism and exhibits strong immunomodulatory properties.^166^ Enormously elevated levels of AFMK were found in the CSF and blood of patients with meningitis,^167^ which suggests a certain role for AFMK in the process of brain inflammation. In addition, alterations of organic acids, isoprostanes, and prostaglandins^112^ and abnormal levels of acetate^153^ were detected in patients with AD.

Overall, metabolomic analysis supports the use of blood‐based metabolites as biomarkers for the prediction of AD, although metabolic biomarker‐based tests still have limited sensitivity and specificity. Hence, it is possible that development of a novel method for the analysis of blood‐based metabolites to improve the accuracy of potential metabolic biomarkers could identify ideal biomarkers for the prognosis and diagnosis of AD. Additionally, a substantial improvement in metabolomic exploration of blood to identify biomarkers for AD diagnosis was made possible by innovations in analytical instrumentation, particularly in the innovative mass spectrometers.

## 5 COULD IDENTIFICATION OF BLOOD‐BASED BIOMARKERS USING A COMBINATION OF PROTEOMICS AND METABOLOMICS BE USED TO PREDICT AD?

**FIGURE 2:**
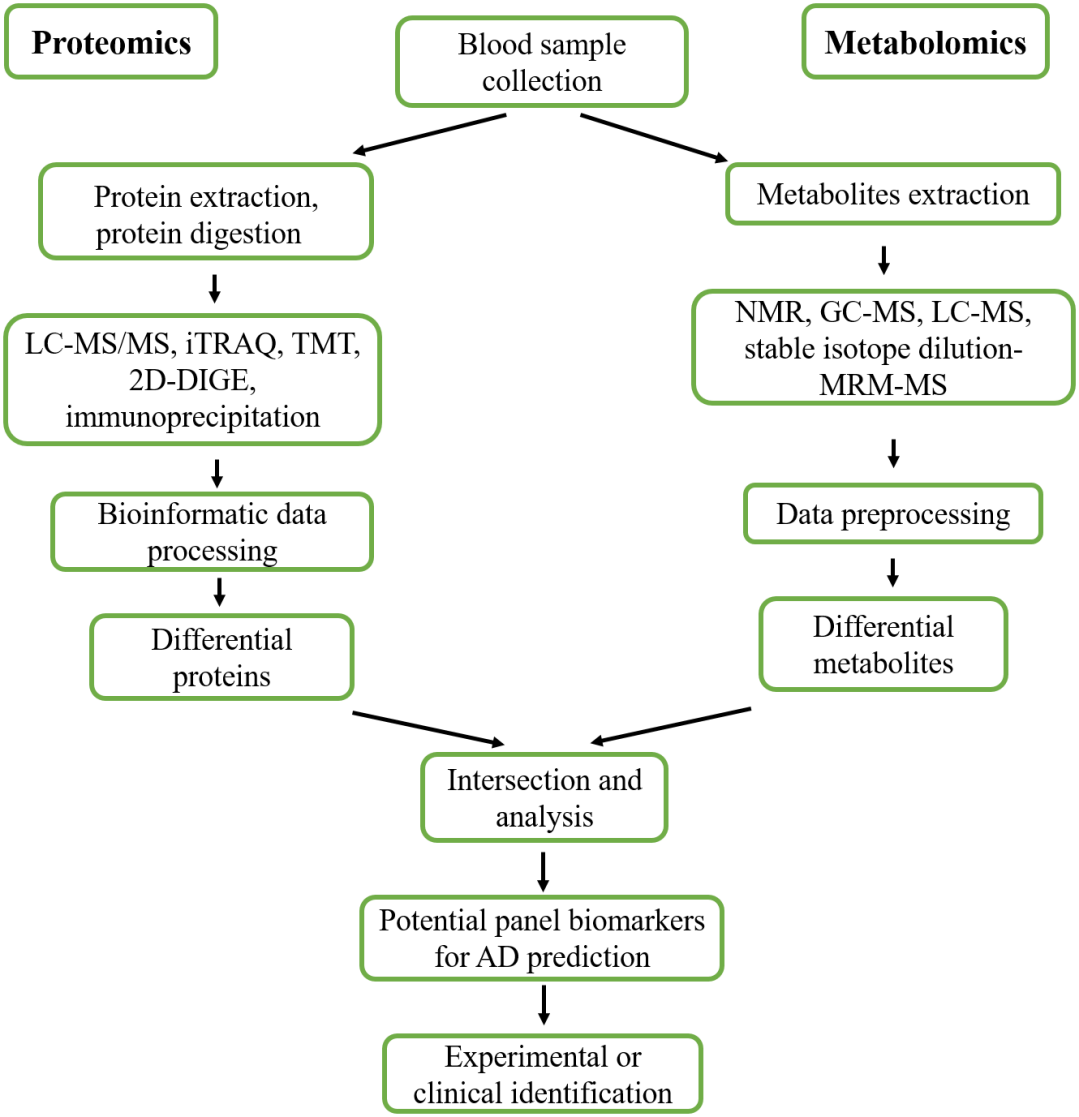
Flowchart shows the identification of putative panel biomarkers using the integration of proteomics and metabolomics for Alzheimer's disease prediction.

Advances in omics platforms are generating a large volume of data from patients with AD and healthy individuals of the same age. Therefore, it is possible that the application of omics will allow a relevant advance in the identification of blood‐based biomarkers for the prognosis and diagnosis of AD because changes at the molecular level can be detected with high sensitivity, specificity, and accuracy before clinical characteristics appear. To effectively monitor AD processes, the combination of proteomics and metabolomics refers to the statistical analysis of large amounts of data at various molecular levels, which verify and complement each other, to achieve a comprehensive understanding of the core mechanisms of molecular biological changes and screen out major biomarkers for in‐depth experimental analysis and clinical application.^168^ Figure 2 depicts a multi‐network approach using proteomics and metabolomics that could identify and verify novel and ideal biomarkers in the blood for prognosis of AD and could be used to develop a reliable prognostic and diagnostic approach and potential therapeutic targets, considering the complex physiopathology of AD.

Combined proteomics and metabolomics analysis reveal the alteration of a certain pathway‐related protein and metabolites in the blood of subjects with AD, which could be as for promising biomarkers for prediction of AD (Table 4). For example, clusterin is one of the main apolipoprotein, which is involved in the transport of cholesterol and phospholipid,^169^ and thus plays a dominant role in lipid metabolism. Importantly, clusterin and cholesterol were increased in blood of AD patients and significantly associated with baseline prevalence and severity of AD.^135^, ^170^, ^171^ These results robustly indicate that clusterin and cholesterol could be developed as an effective blood‐based biomarkers for the prediction of AD. Another panel detected by proteomics and metabolomics is ApoC3 and total cholesterol. ApoC3, the main lipoprotein of low‐density lipoprotein cholesterol, has been shown lower levels, and total cholesterol was increased in blood of subjects with AD, suggesting that abnormal lipid metabolism was in AD procession. Therefore, ApoC3 and total cholesterol could be a novel blood‐based biomarker for AD diagnosis. Although these potential blood‐based signatures involved in lipid metabolism pathway might be ideal biomarkers for prediction of AD, these still need be confirmed by a large number of experiments. Furthermore, novel methods for the analysis of a combination of proteomics and metabolomics should be developed to improve the accuracy of the prediction of AD. Although studies that use multi‐network, combined applications of proteomics and metabolomics to identify biomarkers for AD prediction remain limited, the development of multi‐network analyses for identification of accurate and sensitive molecules can reveal protein‐ and metabolite‐specific pathways involved in the etiology and progression of AD. Overall, we provide a novel list of putative targets and pathways with therapeutic potential, including a set of proteins and their metabolites associated with MIC‐AD.

**TABLE 4:** Overview of promising proteomic and metabolic biomarker candidates involved in lipid metabolism pathway for diagnosis of Alzheimer's disease.

Promising biomarker panels	Changes	Related signaling pathways
Clusterin	**↑**	Lipid metabolism pathway
Cholesterol	**↑**	
ApoC3	**↓**	Lipid metabolism pathway
Total cholesterol	**↑**	

Moreover, because some other neurodegenerative disorders such as Down syndrome (DS) and memory dysfunctions exhibit high comorbidity with AD, their blood‐based biomarkers could be developed as biomarker candidates for early diagnosis of AD. For instance, a study showed that the dramatical functional alternation of some genes included *RAB7A*, *NPC2*, *TGF‐β1*, *GAP43*, *ARSB*, *PER1*, *GUSB*, *MAPT*, *GSK3B*, *PTGS2*, *APOE*, *BACE1*, *PSEN1*, and *TREM2* in the blood of patients with memory disorders.^168^ Importantly, *GSK3B*, *PTGS2*, *APOE*, *BACE1*, *PSEN1*, and *TREM2* well‐known genes implicated in AD. Hence, the protein expression of those genes in blood should be detected and analyzed whether those could differentiate patients with AD and thereby develop biomarkers for early detection of AD processes. Evidence has shown that three neuron‐derived exosome biomarkers, including Aβ1‐42, p‐tau181, and p‐tau S396, were significantly elevated in the subjects with DS compared to control subjects (*p* < 0.0001, *n* = 37 for control participants, and *n* = 47 for participants with DS).^168^ The levels of Aβ1‐42 and p‐tau S396 were increased in DS participants with dementia (*p* < 0.01), indicating that could Aβ1‐42 and p‐tau S396 be useful for AD prediction. However, whether blood‐based p‐tau S396 might predict AD processes needs to be confirmed. Plasma p‐tau181 has almost been shown to be an independent biomarker for AD prediction.

Although we have summarized some blood‐based biomarker candidates, there are still many challenges of biomarkers for the clinical application of AD. Before a biomarker is used in the clinic for AD prediction, several phases must be completed. First, the validation of a biomarker should be focused on in both a research setting and a clinical setting. For instance, the assay development and validation of p‐tau217 as a biomarker for AD prediction has almost been completed.^172^ Second, the biomarker results from a research setting to a clinical setting must be consistent at the level of individual patients, and preanalytical sample handling effects of blood‐based biomarkers should be results from the standardized procedure. Standardization of procedures—ranging from acquisition, handling, and storage of bio‐samples, through assay procedures, together with rigorous documentation—is critical. Third, the assay methodology is important and includes determining sensitivity, cross‐reactivity, and test–retest reliability. Fourth, clinical evaluation of a biomarker for AD prediction should establish large databases, containing the biomarker concentrations and characteristics of individual patients. Finally, the cut‐points based on the evaluation of the assay results in unselected patients will be defined. Thereafter, a clinical biomarker will accelerate drug development for AD therapeutics.

## 6 CONCLUSIONS AND FUTURE DIRECTIONS

The field of blood‐based biomarkers for AD prediction has made rapid progress during the last decade. This may be due to technological advances in sensitive and precise assays, such as omics analyses. The most robust blood‐based biomarkers summarized in this review, including clusterin and cholesterol, ApoC3, and total cholesterol detected by both proteomics and metabolomics, can reliably reflect neurodegeneration in AD. The above blood‐based biomarkers are likely to be valid for AD prediction after extensive and routine analyses in more research studies including longitudinal studies and clinical assessments. These new studies will need to have larger sample sizes. Afterward, there are still several challenges to be addressed on the blood‐based biomarker validity for AD prediction. First, the biomarker validity should be demonstrated in diverse cohorts. Furthermore, there should be analytical standardization for valid biomarker results. A blood‐based biomarker that not only reliably reflects cerebral Aβ and tau pathologies like CSF biomarkers but also independently represents AD pathology in the diverse study population has huge potential as a frequent disease monitoring in clinical and therapeutic settings.

In sum, this review not only provided an overview of recent advances in blood‐based biomarkers but also analyzed potential blood‐based biomarker panel for AD prediction. We summarized current research progress, challenges, and future directions of blood‐based biomarkers for early diagnosis of AD.

## AUTHOR CONTRIBUTIONS

Yun Dong and Xun Song equally contributed to this work. Yun Dong and Zhendan He conceived the project. All authors contributed to the writing the manuscript. All authors read and approved the final manuscript.

## FUNDING INFORMATION

This work was supported by the National Natural Science Foundation of China (31700932), Shenzhen Science and Technology Innovation Council for Basic Research (JCYJ20210324093610027), the Major Project of Guangdong Education Department for Foundation Research and Applied Research (2022ZDZX2062), and the Natural Science Foundation of the Education Department of Guangdong Province (2023KTSCX126).

## CONFLICT OF INTEREST STATEMENT

The authors declare that they have no conflict of interest to disclose.

## Data Availability

All data used to support the findings of this study are included within the article.
